# A Label-Free Proteomic Analysis on Competent Larvae and Juveniles of the Pacific Oyster *Crassostrea gigas*

**DOI:** 10.1371/journal.pone.0135008

**Published:** 2015-08-06

**Authors:** Pin Huan, Hongxia Wang, Baozhong Liu

**Affiliations:** Key Laboratory of Experimental Marine Biology, Institute of Oceanology, Chinese Academy of Sciences, Qingdao, China; Pacific Northwest National Laboratory, UNITED STATES

## Abstract

Current understandings on the molecular mechanisms underlying bivalve metamorphosis are still fragmentary, and a comprehensive description is required. In this study, using a large-scale label-free proteomic approach, we described and compared the proteomes of competent larvae (CL) and juveniles (JU) of the Pacific oyster, *Crassostrea gigas*. A total of 788 proteins were identified: 392 in the CL proteome and 636 in the JU proteome. Gene Ontology analysis of the proteome from each sample revealed active metabolic processes in both stages. Further quantitative analyses revealed 117 proteins that were differentially expressed between the two samples. These proteins were divided into eight groups: cytoskeleton and cell adhesion, protein synthesis and degradation, immunity and stress response, development of particular tissues, signal regulation, metabolism and energy supply, transport, and other proteins. A certification experiment using real-time PCR assay confirmed 20 of 30 examined genes exhibited the same trends at the mRNA and protein levels. The differentially expressed proteins may play roles in tissue remodeling, signal transduction, and organ development during and after metamorphosis. Novel roles were proposed for some differentially expressed proteins, such as chymotrypsin. The results of this work provide an overview of metamorphosis and post-metamorphosis development of *C*. *gigas* at the protein level. Future studies on the functions of the differentially expressed proteins will help to obtain a more in-depth understanding of bivalve metamorphosis.

## Introduction

The Pacific oyster *Crassostrea gigas* has a typical biphasic life cycle with a pelagic larval phase and a benthic adult phase. The transition between the two phases occurs in a relatively short time (generally less than 48 h) during the process of metamorphosis [1,2]. Major morphologic changes in metamorphosis include re-absorption of velum and foot, proliferation and elongation of gills, and the beginning of rapid shell growth [1]. After that, a free-swimming larva develops to a sessile juvenile, which implies a change of habitats. Metamorphosis represents a crucial transition in the development of *C*. *gigas*. Moreover, molluscan metamorphosis is also important in the context of evolution since animal metamorphosis is proposed to arise independently in different clade of animals [3]. A comprehensive understanding on oyster metamorphosis is essential to understand the life of bivalves and the evolution of animal metamorphosis. However, compared to the extensive studies on model organisms such as amphibians, insects, and sea urchins [4,5], current knowledge on molecular mechanisms of oyster metamorphosis is still fragmentary.

It is widely accepted that larvae of many marine invertebrates only initiate metamorphosis in response to particular inducers [6]. For *C*. *gigas*, L-DOPA and catecholamines (epinephrine and norepinephrine) induce metamorphosis [1]. Pharmacological evidence reveals that alpha 1-adrenoceptors mediate metamorphosis [7], but the receptor molecule is unknown. Other genes involved in oyster metamorphosis are also studied individually in the past decade. Tirapé et al. investigated the expression of a set of immune-related genes during ontogenesis of *C*. *gigas* and revealed several genes that potentially function in metamorphosis through regulating extracellular matrix (ECM) remodeling or controlling reactive oxygen species (ROS) level [8]. A transcriptomic study on different developmental stages of the oyster *Crassostrea angulata* revealed some genes potentially important in metamorphosis such as dopamine receptor, adrenergic receptor, and several molecules related to IGF, EGF, and TGF-β pathways [9]. More recently, a genomic investigation of *C*. *gigas* revealed multiple nuclear receptor genes that may play essential roles in metamorphosis [10]. Among studies on individual genes, Yang et al. identified two caspase genes that may function in the degradation of velum and foot during metamorphosis of *C*. *angulata* [11].

Each of the above-mentioned studies revealed some aspects of oyster metamorphosis, however, the information is still fragmentary. In addition, these studies are conducted on the genomic DNA or mRNA levels, and research at the protein level is lacked. Considering the various manners of post-transcriptional and post-translational gene regulations [12,13], research at the protein level is needed to verify previous speculations. Proteomic analysis is proved to be a pivotal approach for studies on animal metamorphosis. It has been applied in many marine invertebrates such as polychaetes [14,15], barnacles [16,17], and bryozoans [15,17]. These studies reveal various proteins that may function in metamorphosis, and provide comprehensive information on the underlying molecular mechanisms. For the studies on oyster metamorphosis, a proteomic analysis would complement and expand current knowledge.

In the present study, we performed a comparative proteomic analysis on competent larvae (the fully developed larvae to perform metamorphosis) and juveniles of *C*. *gigas* using a high-throughput label-free proteomic approach. With the support from the whole genome sequence of the species [18], more than seven hundreds of proteins were identified including 117 differentially expressed proteins. These results provide an overview on oyster metamorphosis and also provide bases for future studies on the functions of the proteins that we identified. Note that a free-swimming larva must settle (to cement to an appropriate substratum) before performing metamorphosis, and the two processes are discriminated from each other traditionally [1,7,19]. However, we use the term “metamorphosis” here to refer to the whole process that a competent larva transits into a sessile juvenile because we do not distinguish among particular events during this process.

## Material and Methods

### Larval culture and sample collection

Artificial fertilization was conducted as described previously [20], where three male and nine female adult oysters were used as parents to avoid too low genetic diversity. The larvae of *C*. *gigas* were cultured in a 1000-litre tank with constant aeration. The seawater was renewed daily and the larvae were fed with *Isochrysis galbana* six times per day. The temperature of seawater ranged from 20°C to 24°C and the pH is 7.8 to 8.0. The competent larvae were collected at 35 days post fertilization (dpf), when approximately 50% of the larvae developed eye spots and started to crawl. Wavy polyethylene sheets (length × width: 50 × 50 cm) were applied as bases at 36 dpf to promote metamorphosis as described previously [21]. The juveniles were collected at 50 dpf by scraping them from the bases carefully. All samples were immediately frozen in liquid nitrogen and stored at -80°C until protein extraction.

### Protein extraction, quantification, and quality control

The two samples (competent larvae (CL) and juveniles (JU)) were used in proteomic analysis. Protein extraction, reduction, and acetylation were conducted as described previously [22] except that a modified lysis buffer (7 M urea, 2 M thiourea, 4% CHAPS, 40 mM tris-HCl, pH 8.5) was used. Protein was quantified using the 2-D Quant Kit (GE Healthcare). Thirty micrograms of each protein sample was submitted for 12% SDS-PAGE for quality control. We prepared one protein sample from each developmental stage given that they both represented a pool of tens of thousands individuals.

### Protein digestion and LC-MS/MS

One hundred micrograms of each protein sample was digested by trypsin as described previously [22]. The resultant peptide samples were desalted by a Strata X C18 column (Phenomenex) and re-suspended in 200 μL volumes of re-suspending buffer (2% acetonitrile, 0.1% formic acid) to yield a concentration of approximately 0.5 μg/μL. After centrifuging at 20000 g for 10 min, 10 μL of supernatant was subjected to liquid chromatography with tandem mass spectrometry (LC-MS/MS) analysis. High performance liquid chromatography (HPLC) was conducted using an LC-20AD nanoHPLC (Shimadzu), which was coupled online with an LTQ Orbitrap Velos (Thermo) used for MS/MS analysis. All parameters for the LC-MS/MS analysis were set consistent with Nie et al. (2013). Each peptide sample was analyzed twice as technical replicates.

### Data analysis of tandem mass spectra

Tandem mass spectra were searched against the *C*. *gigas* UniProt database (25,981 proteins) using Myrimatch [23]. A decoy database containing reverse sequences of the proteins was also used. The resultant peptides were assembled into protein groups using IDPicker with a false discovery rate (FDR) of 5% [24]. Protein groups were minimized to contain only one protein [25]. Only proteins with at least two distinct peptides were considered reliable.

Quantitative analysis was conducted based on spectra count. The fold change (FC) of each protein was assessed by comparing the log_2_ spectra count in JU to that in CL (log_2_ (JU/CL)). The mean and standard deviation (SD) of FC values were used as the criteria for differential analysis. The proteins with FCs falling out of the range of one SD of the mean were considered differentially expressed [25]. We eliminated proteins with low expression because they might cause false positive results. The highest spectra count of one protein among the four runs (two runs per sample) was defined to be H-sc. The proteins with H-sc less than ten were considered to have low expression and were eliminated. In addition to the proteins detected in both stages, proteins only detected in a single stage were also considered as differentially expressed, once their H-sc were ten or more.

### Gene ontology analysis

Gene ontology analysis was conducted on whole proteins that were identified from each sample using UniProt GOA (*p* < 0.05).

### Real-time PCR

Thirty genes were selected to measure their mRNA expression. The cDNA sequences of the genes were retrieved from the whole genome sequence database of *C*. *gigas* (OysterDB, http://www.oysterdb.com) and the primers are provided in Table 1. A set of independent samples were used in the mRNA expression analysis. The larvae were cultured in seawater with a slightly higher temperature (24–25°C) and grew faster. The competent larvae merged at 27 dpf and juveniles were collected at 37 dpf, when the polyethylene bases were applied for 10 days.

**Table 1:** The primers of 30 interested genes and reference genes.

UniProt Accession/gene	OysterDB Accession	Sense Primer	Anti-sense Primer	Efficiency
K1QFS1	CGI_10006107	GAGGATGGCTGTGAGTTGAACTGTGT	ATGCTGGCTGTGGTGGCTGAGA	1.01
K1R3E8	CGI_10023717	AACGACAGGCAGGCTACCACTACT	ACTTGCTCCAATGTCCGTCAACTTCA	1.01
K1PDA4	CGI_10008455	GGTCAAGAAGGATGGTGAATGCTGTCT	AGCCGCAGGAAGAATTGAGTAGGTG	0.90
K1R0Q6	CGI_10026821	TGTCATCCAGGCTTCTACCGCTACC	ACTCACACTGTCCACTCAACTGTTCAC	1.02
K1PTA3	CGI_10009194	TCTGCATGGTATCTGACGGAGAACG	TCTGGCTCTTTGCTACGGTTGATGA	0.91
K1QWZ6	CGI_10023347	TTCTACCTGCTCTTCCTGGCTGTCA	TGTACTTGTTGATAGCGTCGTCGTAGC	0.94
K1R6E6	CGI_10025660	GTGATGGCGACAATGACTGTGGAGAT	GAGGCGGAGCAATGTTAATGGATGGA	0.95
K1PPU7	CGI_10012348	CAATCGTGTTCCTATCTGTGGCTCCT	ACAAGTCAACTCCAGGCGTGTTCC	1.01
K1PZ82	CGI_10003195	AACTTGATAGCGTCACCTCCTTCTTCG	CCTGTTGTTCTTTCTGTTGTCGTAGCA	0.97
K1QHM2	CGI_10009177	ACCTGTTCCGTGAGCCCGAGAA	GCTGCGAGACAGAGATGGAATCCTAC	0.94
K1Q4S5	CGI_10025806	ACACCAGATGGAGTGCCAGTTGATAGA	TGTCTGCTGTGGAGTCGCTGTCA	0.93
K1R289	CGI_10013230	GCAAGGCATTGGACGAAGCACTG	TGACCTGGATCGCTTGGACATCAAC	0.99
K1QQV6	CGI_10022006	GCTTCTGTTGTCACTGGTCCTTGTTG	ATGTTGAGATCGGCGTCATCACTGAA	0.93
K1R3T3	CGI_10019268	GTCCACCGAACAGCTACAACAGATGA	AAGATTCCAGGCAACATTTCAGCGATC	0.99
K1PM66	CGI_10000595	CCTCATCAAGAGCCTGAAGGAACCA	GGACATCTTTCTACAGAGCACCCAACT	0.92
K1PQP2	CGI_10004474	AGTGAAGAGGAGGAGGAGGAAGAAGAA	CTTTGTCTACGCTGCTCGCAATGTTT	0.97
K1Q2L2	CGI_10009164	CCTCATCAAGAGCCTGAAGGAACCA	GGACATCTTTCTACAGAGCACCCAACT	0.96
A7M7T7	CGI_10008877	GCTGGAGCCAGAGTTCAAGAAGAGG	GCCAATGATGAATACCGCACGACAT	0.93
K1R7F8	CGI_10027917	AGCATTGCCTGTATCTCTGTCAACGAT	TCTCCGCATGTATCTGCCAACATTCTA	1.06
K1PWQ2	CGI_10018067	AGATCACCAGCCTAAGAGCCGAGAT	GCTACGCTTGCTACTATACCACGAACA	1.01
K1Q2X5	CGI_10021682	AGGAGAGGAACATCATGGACAGTGGAT	TCGTAGAAAGCGGAATCAGCATAGTGT	0.97
K1QRU8	CGI_10025109	CGGTGGCTGGTTAGAGAAGAACAAGG	AGATGGTCTGGAAGGAAGCGGACTT	0.97
K1Q801	CGI_10001587	TTGCCATGTTTGACAAAGACGGAAAGG	TATGCCTCCTCTGAGCTGCTACCC	0.95
K1PAG1	CGI_10005381	AAGTTCCGCTCCATTCCTCCATTCAG	CGCCGAGTCCATCAGTTCTACCATC	0.98
K1PX83	CGI_10017921	CTCTCCACATCCATCAACTCCACCAA	GATCTCCACCTCCAGCTCCTCGTA	1.02
K1QM96	CGI_10015381	ATCTATTGGTCGTGTTGTGTTGGGTGA	CCGTAATCCAGCACTCGTTGTTATTGA	0.98
K1R712	CGI_10018984	CAAGAAGATGATGTTGAGGAGGCTGGA	GGTGTGCTGGTCTGTGTGGGTTT	0.96
K1R6J8	CGI_10017544	CGAATGAACGACCACTGCAACAAGTC	TGTCTGTCTGTCCAGGCTGTAGGC	0.95
K1PHQ7	CGI_10012352	GACTCTTGTTACCGTCCTGTGAACTGT	GCTTGGATAGGTCTCTGTTGGCTTCTT	0.90
K1QX16	CGI_10016965	GCGGTGCTGTCGTCATGTGTCA	AGATCCATCTAAGTCGGGTTGGGTCAA	1.05
*elf* (reference)	CGI_10012474	CGAGAAGGAAGCTGCTGAGATGG	ACAGTCAGCCTGTGAAGTTCCTGTA	0.90
*gapdh* (reference)	CGI_10010974	AGAACATCGTCAGCAACGCATCC	CCTCTACCACCACGCCAATCCT	0.96
*arf-1* (reference)	CGI_10017557	TCAGGACAAGATCCGACCACTGT	GCAGCGTCTCTAAGTTCATCCTCATTA	1.02

RNA was extracted using the E.Z.N.A. total RNA kit II (Omega Bio-Tek, USA) and reverse-transcribed using the Primerscript RT reagent Kit with gDNA Eraser (Takara, China). Real-time PCR was conducted in triplicate in a 10 μL volume containing 1X SuperReal PreMix Plus (Tiangen, China), 0.25 μM of each primer, and 0.25 μL of cDNA. Three housekeeping genes, *elf*, *gapdh*, and *arf-1* were used as internal references. Among the three genes, *elf* was widely used in studies on *C*. *gigas* development [26] while *gapdh* and *arf-1* were revealed to be stably expressed in adult tissues [27]. The geometric means of their Ct values were used for normalization as suggested by Vandesompele et al. [28], and the relative expression levels of the genes were calculated using the 2^-ΔΔCt^ method [29]. The expressions of genes in the two stages were compared using the Student’s *t*-test.

## Results and Discussion

### LC-MS/MS and Protein identification

**Fig 1:**
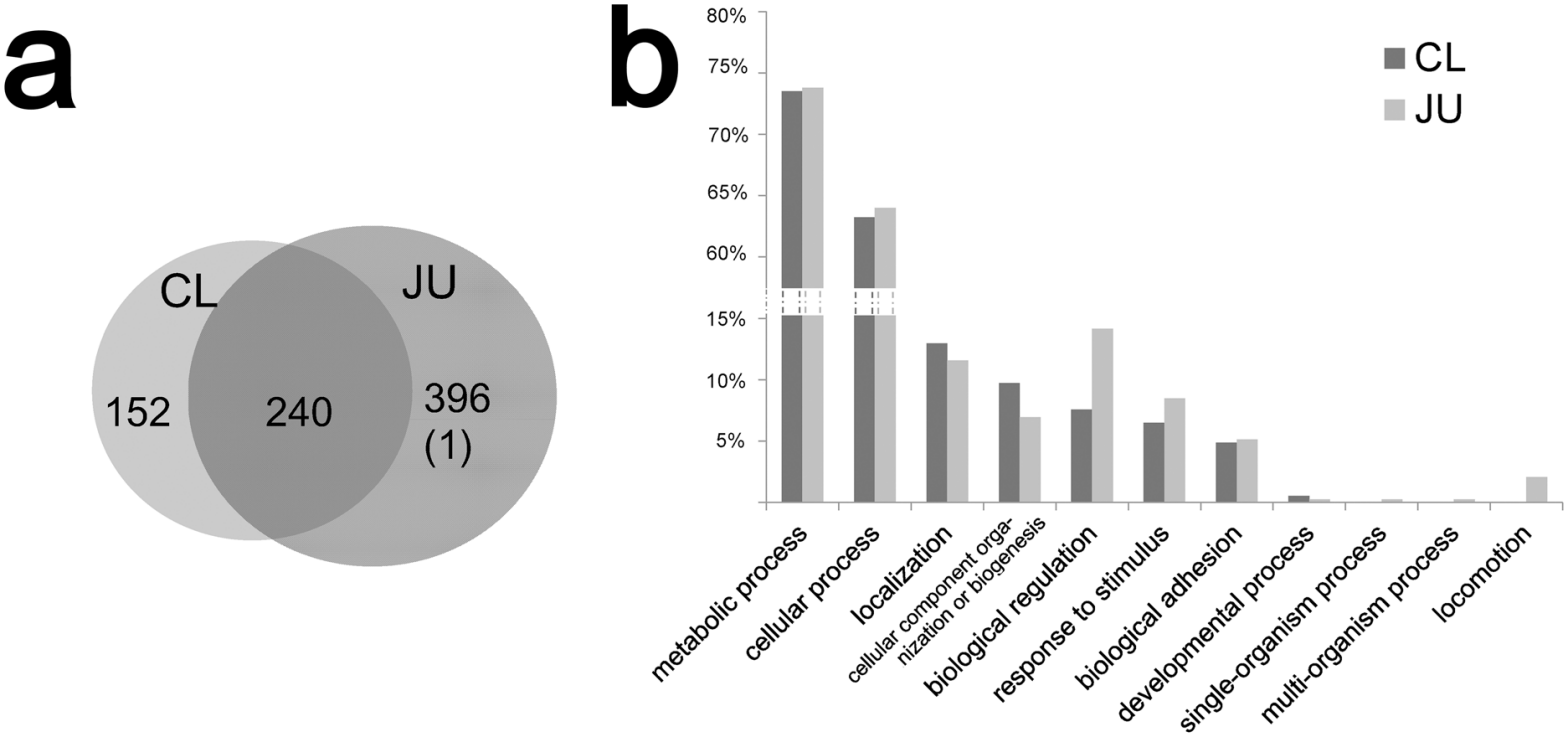
Protein identification and GO analysis. a. A Venn diagram showing the numbers of proteins identified from each sample. The number in the bracket indicates a hit from the decoy database. b. Results of GO analysis (the "biological process" category). More information of GO analysis is provided in S2 Table.

Totally 2558/2524 and 4416/4313 filtered spectra were obtained in two runs of CL and JU, respectively. These spectra were used to search against *C*. *gigas* protein database and then assembled into protein groups. The statistics of protein identification were shown in Table 2. A total of 788 proteins were identified, where 392 and 636 proteins were identified from CL and JU, respectively (S1 Table). In JU, one hit was retrieved from the decoy database (K1PYK0_CRAGI), and it was eliminated from the following analyses. Two hundred and forty proteins were common in both samples. Among the remaining proteins, 152 were unique to CL and 396 were unique to JU (Fig 1a). At least 81% of proteins identified in a run were also observed in its technical replicate (Table 2), revealing good reproducibility between technical replicates.

**Table 2:** Statistics of protein identification.

Run	Filtered Spectra	Distinct Peptides	Distinct Matches	Protein Groups	Proteins	Repeated proteins in technical replicate	Repeated proteins in another sample
**CL-1**	2558	1198	1370	330	349	81.95%	61.60%
**CL-2**	2524	1159	1343	314	329	86.93%	62.61%
**JU-1**	4416	2105	2364	543	561	81.46%	38.50%
**JU-2**	4313	2052	2310	514	531^a^	85.74%	41.84%

More details were provided in S1 Table.

^a^: include one hit from the decoy database.

More filtered spectra were obtained from JU than CL, which resulted in a greater number of protein identifications. It is not likely caused by technical reasons because same amount of the two samples were loaded and the two technical repeats of each sample exhibited similar trends. This result may be caused by different compositions of the two proteomes. The different protein compositions, e.g. different proteins or different types of post-translational modifications [13], would have brought different characters of the two proteomes, such as hydrophobicity and distributions of cleavage sites of trypsin. These different characters might cause different behaviors of the two samples in trypsin digestion, liquid chromatogram, and mass spectrometry, and finally caused the different numbers of mass spectra.

### Characterization of the proteomes of CL and JU

Gene ontology (GO) analysis was conducted to provide an overview of the proteomes of the two samples. In the category of "biological process", 185 and 389 proteins from CL and JU were assigned into particular categories, respectively. Fig 1b shows the GO terms at level 2, which were similar in the two proteomes. Besides the two categories "metabolic process" and "cellular process" that contained most proteins (over 60%), other major categories include "localization", "biological adhesion", "cellular component organization or biogenesis", and "biological regulation". Notably, several proteins (6.49% and 8.48%) of the two samples clustered into the category "response to stimulus", reflecting the proteins related to immunity and stress response are possibly active in the two stages.

Though there were apparently similar results when considering the general GO terms, it is a little arbitrary to simply conclude the proteomes of the two stages are similar. It is possible that GO terms at lower levels may differentiate. On the other hand, because we conducted a differential analysis in the following section, we will not compare in detail the results of the GO analyses of the two proteomes. The results of the GO analyses, including both general GO terms and the terms at lower levels, are provided in S2 Table, which we expect to provide support for related research.

### Quantitative analysis

**Fig 2:**
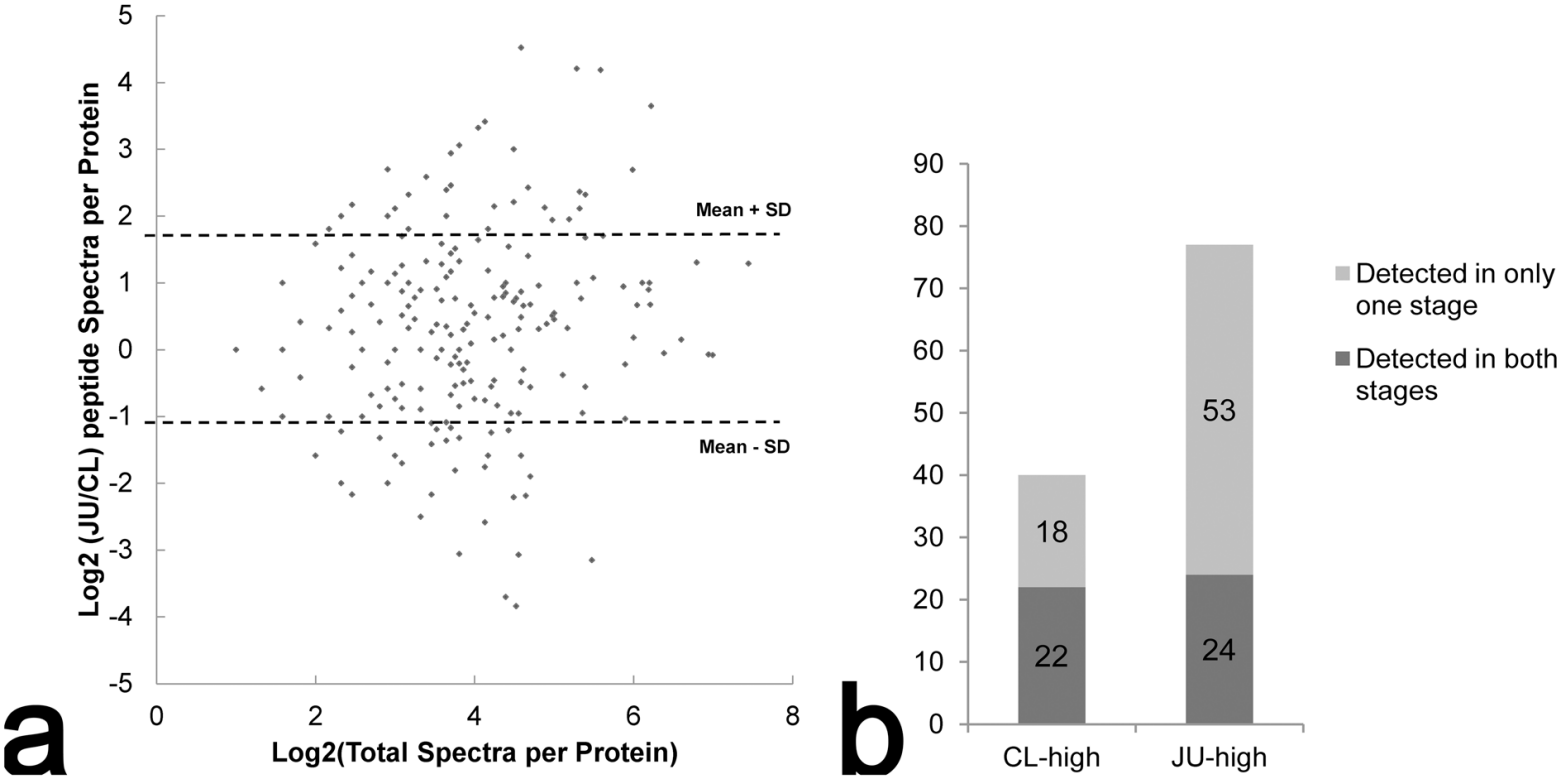
Quantitative analysis between the two developmental stages. a. The scatter plot distributions of the FCs of the 240 proteins that were detectable in both stages. The Y axis represent FC and the X axis represents the log_2_ transformation of total spectra from the four runs. The range of one SD of the mean is indicated by dashed lines. Those proteins fell out of the range are candidate differentially expressed proteins. b. The distributions of the 117 differentially expressed proteins. All proteins have the H-sc of at least ten.

Spectra count of every protein was used in quantitative analysis. For the 240 proteins that were detected in both samples (Fig 1a), the FCs (Log_2_ (JU/CL)) varied from -3.84 to 4.52, with the mean and SD of 0.32 and 1.39, respectively. Fig 2a shows the scatter plot distributions of the 240 proteins, where the FC (Y axis) and the log_2_ transformation of total spectra of all runs (X axis) are exhibited. Forty six proteins with H-sc of at least ten were selected to be differentially expressed proteins from 71 proteins whose FCs fell out of the range of one SD of the mean. For the proteins only detectable in one stage, 71 proteins with H-sc of at least ten were selected. In total 117 differentially expressed proteins were obtained, including 40 highly expressed in CL (CL-high, 22 detected in both samples and 18 detected only in CL) and 77 highly expressed in JU (JU-high, 24 detected in both samples and 53 detected only in JU) (Fig 2b).

Initially we attempted to conduct a gene ontology analysis to associate the differentially expressed proteins with particular biological processes. However, no significant result was obtained possibly because the proteins were functionally diverse and the total number of proteins was relatively small for gene ontology analysis. Therefore, we manually divided the proteins into eight groups according to their potential functions based on sequence homology (Table 3, S3 Table).

**Table 3:** A part of differentially expressed genes/proteins between CL and JU.

Accession	FC (protein)	FC (mRNA)^a^	Annotation	Description
**Cytoskeleton and cell adhesion**				
K1QFS1	-3.06 (↓)	-1.54 (↓), *p*<0.05	Collagen alpha-6(VI) chain	ECM
K1R3E8	-1.90 (↓)	n.v. (-), *p*>0.05	Collagen alpha-5(VI) chain	ECM
K1PDA4	-3.07 (↓)	-0.95 (↓), *p*<0.05	Collagen alpha-4(VI) chain	ECM
K1R0Q6	2.32 (↑)	1.10 (↑), *p*<0.05	Laminin subunit alpha	ECM
K1PTA3	JU-specific (↑)	1.03 (↑), *p*<0.05	Collagen alpha-4(VI) chain	ECM
K1QWZ6	1.95 (↑)	n.v. (-), *p*>0.05	Dolichyl-diphosphooligosaccharide—protein glycosyltransferase subunit 1	ECM
K1R6E6	1.94 (↑)	1.43 (↑), *p*<0.05	Basement membrane-specific heparan sulfate proteoglycan core protein	ECM
K1PPU7	JU-specific (↑)	8.95 (↑), *p*<0.05	Hemicentin-1	ECM
K1PZ82	JU-specific (↑)	5.30 (↑), *p*<0.05	Collagen alpha-3(VI) chain	ECM
K1QHM2	3.00 (↑)	0.23 (↑), *p*<0.05	Dolichyl-diphosphooligosaccharide—protein glycosyltransferase subunit 2	ECM
K1Q4S5	JU-specific (↑)	1.49 (↑), *p*<0.05	Cadherin-87A	ECM
K1R289	JU-specific (↑)	3.73 (↑), *p*<0.05	Collagen alpha-3(VI) chain	ECM
K1QQV6	JU-specific (↑)	-0.49 (↓), *p*<0.05	Collagen alpha-1(XII) chain	ECM
K1PSE7	-1.75 (↓)	not examined	Outer dense fiber protein 3	cytoskeleton component
K1QT91	-2.21 (↓)	not examined	LIM domain and actin-binding protein 1	cytoskeleton component
K1QWP8	CL-specific (↓)	not examined	Actin-2	cytoskeleton component
K1Q821	CL-specific (↓)	not examined	Actin, cytoplasmic	cytoskeleton component
K1QGK1	CL-specific (↓)	not examined	Fibrillin-2	involved in cell adhesion
K1QCL6	-1.42 (↓)	not examined	Proteasomal ubiquitin receptor ADRM1	involved in cell adhesion
K1QXX7	2.37 (↑)	not examined	Myosin heavy chain, non-muscle (Fragment)	cytoskeleton component
K1RH58	JU-specific (↑)	not examined	Alpha-actinin, sarcomeric	cytoskeleton component
K1PE57	2.21 (↑)	not examined	Severin	cytoskeleton component
K1QFR9	JU-specific (↑)	not examined	Spectrin beta chain	cytoskeleton component
K1RBG6	JU-specific (↑)	not examined	Actin-1/3	cytoskeleton component
K1Q086	JU-specific (↑)	not examined	Ankyrin-2	cytoskeleton-binding protein
K1QB61	3.65 (↑)	not examined	Protocadherin Fat 4	involved in cell adhesion
K1QR13	JU-specific (↑)	not examined	Disks large-like protein 1	involved in cell adhesion
K1QMS2	JU-specific (↑)	not examined	Cadherin EGF LAG seven-pass G-type receptor 3	involved in cell adhesion
**Protein synthesis and degradation**				
K1R3T3	-1.58 (↓)	-0.62 (↓), *p*<0.05	Transcription factor BTF3-like protein 4	transcription factor
K1PM66	-1.17 (↓)	n.v. (-), *p*>0.05	60S ribosomal protein L12	ribosomal protein
K1PQP2	-1.32 (↓)	n.v. (-), *p*>0.05	Nucleolin	ribosomal protein
K1Q2L2	-1.17 (↓)	n.v. (-), *p*>0.05	60S ribosomal protein L12	ribosomal protein
K1RNZ9	CL-specific (↓)	not examined	Glucosidase 2 subunit beta	involved in glycoprotein folding and modification
K1RJ91	CL-specific (↓)	not examined	Ubiquitin-associated protein 2	involved in protein degradation
K1QNZ7	CL-specific (↓)	not examined	Ubiquilin-1	involved in protein degradation
K1PNQ5	JU-specific (↑)	not examined	Heat shock protein HSP 90-alpha 1	involved in protein folding and stress response
K1Q7T5	JU-specific (↑)	not examined	Protein disulfide-isomerase A3	involved in protein folding
K1QX26	2.46 (↑)	not examined	Endoplasmin	involved in protein folding and stress response
K1QBF7	2.39 (↑)	not examined	Hypoxia up-regulated protein 1	involved in protein folding and stress response
K1RCT9	JU-specific (↑)	not examined	Carboxypeptidase B	involved in protein modification
K1QR72	JU-specific (↑)	not examined	Dipeptidyl-peptidase 3	involved in protein modification or degradation
K1R866	1.81 (↑)	not examined	Puromycin-sensitive aminopeptidase	involved in protein degradation
**Immunity and stress response**				
K1R7F8	-3.15 (↓)	-1.41 (↓), *p*<0.05	Peroxiredoxin-5, mitochondrial	anti-oxidant enzyme
A7M7T7	-1.17 (↓)	n.v. (-), *p*>0.05	Non-selenium glutathione peroxidase	anti-oxidant enzyme
K1QE94	JU-specific (↑)	not examined	Alpha-N-acetylgalactosaminidase	immune-related protein
**Particular tissue development**				
K1QM96	-1.36 (↓)	-7.68 (↓), *p*<0.05	Chymotrypsin B	digestive enzyme
K1PWQ2	2.13 (↑)	0.7 (↑), *p*<0.05	60 kDa neurofilament protein	neuron-specific protein
K1Q2X5	CL-specific (↓)	-1.24 (↓), *p*<0.05	Synaptopodin-2	neuron-specific protein
K1PP20	2.00 (↑)	not examined	Mammalian ependymin-related protein 1	neuron-specific protein
K1PWP8	JU-specific (↑)	not examined	Echinoderm microtubule-associated protein-like 1	neuron-specific protein
K1QRU8	2.69 (↑)	0.31 (↑), *p*<0.05	Myosin heavy chain, striated muscle	muscle-specific protein
K1Q801	-1.81 (↓)	-0.65 (↓), *p*<0.05	Myosin regulatory light chain A, smooth adductor muscle	muscle-specific protein
K1QTC1	JU-specific (↑)	not examined	Paramyosin	muscle-specific protein
K1REY2	JU-specific (↑)	not examined	Dysferlin	muscle-specific protein
K1PAG1	4.19 (↑)	-0.79 (↓), *p*<0.05	Dynein beta chain, ciliary	cilia-specific protein
K1PX83	JU-specific (↑)	n.v. (-), *p*>0.05	Dynein heavy chain 5, axonemal	cilia-specific protein
K1QHI6	JU-specific (↑)	not examined	Dynein heavy chain 5, axonemal	cilia-specific protein
K1QK11	JU-specific (↑)	not examined	Dynein heavy chain 7, axonemal	cilia-specific protein
**Signal regulation**				
K1R712	CL-specific (↓)	0.38 (↑), *p*<0.05	Transforming growth factor-beta receptor-associated protein 1	involved in TGF-β signaling pathways
K1R6J8	JU-specific (↑)	JU-s*p*ecific (↑)	Wnt inhibitory factor 1	involved in Wnt signaling pathways
K1PHQ7	JU-specific (↑)	8.76 (↑), *p*<0.05	Follistatin-related protein 4	involved in TGF-β signaling pathways
K1QX16	JU-specific (↑)	3.84 (↑), *p*<0.05	Tyrosine-protein phosphatase Lar	signal regulator

We present the major differentially expressed proteins of curiosity in this table. An expanded table is available in S3 Table. The fold changes (FCs) were calculated using the formula: FC = Log_2_ (JU/CL). “↑” and “↓” indicate higher expression in JU and CL, respectively. “–” indicates no significant difference between the two stages (no variation, n.v.).

^a^: The Student’s *t*-test was used to compare the mRNA expressions between the two stages and the difference was considered significant when *p* <0.05.

### mRNA expression of selected genes

We used two pooled samples in proteomic analysis. To confirm the protein differences at the transcript level we analyzed the mRNA expression of 30 differentially expressed key proteins. Twenty genes (66.67%) exhibited the same trends at the mRNA and protein levels (Table 3, S1 File). In addition to the appearance of false positives with one of the methods, the inconsistency between both data sets might also be due to differences between post-transcriptional/translational regulations. In the following we focus on those genes that showed the same trends at the mRNA and protein levels.

### Potential roles of the differentially expressed proteins

Molluscan metamorphosis is important to understand the life and evolution of the species of this phylum. Through a proteomic approach, we identified over a hundred proteins differentially expressed before and after metamorphosis. A certification experiment using 30 genes revealed two thirds of them exhibited the same trends at the mRNA level. We will discuss the potential roles of some differentially expressed proteins of curiosity in transition of the molluscan lifestyle based on homology.

#### Proteins related to cytoskeleton and cell adhesion

"Skeletons" exist inside and outside of cells. Their intracellular, transmembrane, and extracellular components, mainly referring to cytoskeleton, cell adhesion, and ECM, respectively, comprise an important network. This network allows cell motility including cell shaping, cell migration, cell proliferation, and programmed cell death etc. A high level of cell motility in both metamorphosis and post-metamorphosis development may be reflected by the 28 differentially expressed proteins (~24% of total differentially expressed proteins) that are involved in functions of cytoskeleton, cell adhesion, and ECM. These proteins may contribute to the tissue remodeling during metamorphosis, such as degradation of velum and foot and transitions of muscular and nervous systems.

We will discuss more the ECMs due to their potential roles in tissue remodeling and regulating cell fate. Totally 13 differentially expressed ECMs were revealed in proteomic analysis. In real-time assay, we analyzed all these 13 genes and ten of them exhibited the same trends. ECMs provide the base for cells, and therefore they must be remodeled in tissue remodeling of metamorphosis [30,31]. ECM remodeling has been proved essential in metamorphosis of amphibian [31,32] and insects [33]. In *C*. *gigas*, a matrix metalloprotease inhibitor gene is induced before metamorphosis and possibly regulated ECM remodeling in metamorphosis [8]. The changes of ECMs that we observed emphasize the roles of ECM remodeling in metamorphosis of *C*. *gigas*. Another important role of ECMs in animal metamorphosis is regulating cell fate. *In vitro* experiment on intestinal epithelial cells of larval amphibians reveals that the accessibility to ECMs determines whether a cell will undergo apoptosis [34]. We speculate that ECMs may also function in regulating cell fates in metamorphosis of *C*. *gigas*, which certainly requires further confirmation.

#### Proteins related to protein synthesis and degradation

Generally, it is believed that transcription and translation are not necessary in the initial phase of metamorphosis in marine invertebrates. Larvae of a wide range of marine animals, including gastropods, polychaetes, bryozoans, and barnacles, can undergo the initial phase of metamorphosis under treatments of translation and/or transcription inhibitors [17,35,36]. Therefore, it was interesting to observe that four proteins related to transcription or translation, including a transcription factor and three ribosomal proteins, were highly expressed in CL. In the abalone *H*. *asinine*, a ribosomal protein gene is also observed to be highly induced in competent larvae [37]. However, in real-time assay, we revealed that only the transcription factor gene exhibited high mRNA expression in CL. In contrast, mRNA expression of the other three ribosomal proteins genes did not differ significantly between the two stages. Thus far, it still remains unclear whether transcription/translation inhibitors would affect the metamorphosis of *C*. *gigas*. A pharmacological experiment is required to estimate the roles of transcription/translation-related genes in metamorphosis.

#### Proteins related to immunity and stress response

Up-regulation of molecules related to immunity and stress response is a common phenomenon in animal metamorphosis [5]. Among the three differentially expressed proteins related to immunity and stress response, the two CL-high proteins were both anti-oxidant enzymes, including a glutathione peroxidase (GPx) and a peroxiredoxin (Prx). We analyzed the two genes in real-time PCR assay and confirmed one of them (the Prx gene) had significantly higher mRNA expression in CL. Moreover, although it was not revealed to be differentially expressed in the present study, the mRNA of another antioxidant enzyme of *C*. *gigas*, an extracellular superoxide dismutase (EcSOD), was proved to be highly induced during metamorphosis [8]. Given that the expression of anti-oxidant enzymes is tightly regulated to avoid cell damage by ROS, the high expression of these enzymes may indicate that there is elevated ROS stress in CL. In amphibian, elevated ROS stress during metamorphosis is caused by enhancement of mitochondrial respiration under the induction of metamorphosis-inducing hormone (thyroid) [38,39]. High ROS levels may be generated through similar mechanisms in the metamorphosis of *C*. *gigas*, and the anti-oxidant enzymes should play important roles in maintaining homeostasis.

#### Proteins indicating development of specific tissues

The differential expression of proteins that are dominant in specific tissues can indicate changes in them. For instance, the expressions of two tissue-specific genes (tropomyosin and chymotrypsin) are highly relevant to the development of muscles and the digestive system during metamorphosis of the abalone *H*. *rufescens* [40,41]. The changes of muscle- and neuron-specific proteins between CL and JU, such as myosin heavy chain and synaptopodin protein, may reflect the transition of muscular and nervous systems during molluscan metamorphosis [5,41]. In real-time PCR assay, we analyzed two neuron-specific and two muscle-specific genes, and the results confirmed their mRNA expression exhibited the same trends with that of the protein level. Four ciliary dynein proteins were revealed to be highly expressed in JU, indicating an enhancement of ciliated tissues in JU. This seems to be paradoxal because the conspicuous ciliated organ of CL (the velum) vanished in JU. In real-time PCR assay, we analyzed two of them, and the results revealed either no significant difference or a higher expression in CL. Investigations are needed to reveal the changes of ciliated tissues as well as the expression of dynein genes during metamorphosis.

A chymotrypsin protein was observed to be highly expressed in CL and the same trend was observed at the mRNA level. This is consistent with a previous observation that a chymotrypsin gene that is specifically expressed in the digestive system is highly induced during the metamorphosis of *H*. *rufescens* [40]. We speculate that this may reflect a transition of the digestive system in metamorphosis. Moreover, the low expression of the chymotrypsin protein in JU suggests possibly other roles for chymotrypsin in metamorphosis.

#### Proteins related to signal regulation

Proteins related to signaling pathways are often absent in proteomic studies based on gel electrophoresis [20,42] possibly due to the relatively low expression levels of these proteins. With the support of high-throughput proteomic approaches, we successfully detected several differentially expressed proteins related to particular signaling pathways. These include one negative regulator of Wnt pathways only detected in JU, two regulators of TGF-β pathways only detected in each of the two samples, and one tyrosine-protein phosphatase only detected in JU.

Three of the four signal regulators were only detected in JU, suggesting Wnt pathways, TGF-β pathways, and pathways involving tyrosine phosphorylation are active in juvenile development. We analyzed the mRNA expression of the three genes and confirmed their higher expression in JU. These three types of pathways are all canonical signaling pathways that function widely in developmental of animals [43–45]. The three differentially expressed proteins may contribute to the development of multiple organs of JU, such as shells, gills, and muscular and nervous systems.

The one signal regulator that was only detected in CL is a protein associated with TGF-β pathways, which indicate that particular TGF-β pathways may be active in the metamorphosis of *C*. *gigas*. However, when we analyzed its mRNA expression, the results revealed it was expressed in both stages with a higher expression in JU. The inconsistency at the mRNA and protein levels provokes the question of whether this gene participates in *C*. *gigas* metamorphosis. Nevertheless, we still suggest a further investigation on the gene when considering the essential roles of TGF-β pathways in *Drosophila* metamorphosis [46].

#### Other proteins

Other differentially expressed proteins included 21 proteins related to metabolism and energy supply, 15 proteins related to transport, and 19 un-grouped or un-annotated proteins (S3 Table). It is not easy to correlate their expression trends to particular biological processes, which makes it difficult to infer their functions in the transition of lifestyle. However, those proteins may provide hints for future studies. For instance, all the 15 protein related to transport were all highly expressed in JU (S3 Table), indicating active cellular transport after metamorphosis. This may reflect the higher adaptability of sessile juveniles because they have lost the ability to actively avoid environmental stresses such as extreme salinity and ion conditions.

## Conclusions

In conclusion, through comparing the proteomes of competent larvae and juveniles of *C*. *gigas*, we provide an overview on metamorphosis and post-metamorphosis development at the protein level. Quantitative analysis revealed over a hundred differentially expressed proteins between the two stages. A certification experiment confirmed two thirds of 30 interested genes exhibited the same trends at the mRNA and protein levels. Many aspects of the two developmental stages, such as tissue remodeling, stress response, and organ development, are reflected by the differentially expressed genes/proteins. These results will enrich our understanding of molluscan metamorphosis. Furthermore, novel roles are suggested for some proteins, such as chymotrypsin. Our results expanded the knowledge of the molecular mechanisms of molluscan metamorphosis. The supplemental data we provided can provide hints and supports for future studies.

## Supporting Information

S1 File The Results of real-time PCR. (PDF)

S1 Table The results of protein identification. (XLS)

S2 Table The results of GO analysis. (XLS)

S3 Table Details of the differentially expressed proteins. (XLS)
