# Supplementary material for: A Label-Free Proteomic Analysis on Competent Larvae and Juveniles of the Pacific Oyster Crassostrea gigas
Source: PLoS One. 2015 Aug 6;10(8):e0135008. doi: 10.1371/journal.pone.0135008 (PMC4527670; doi:10.1371/journal.pone.0135008)
Supplement: S1 File — (PDF) [file pone.0135008.s001.pdf]

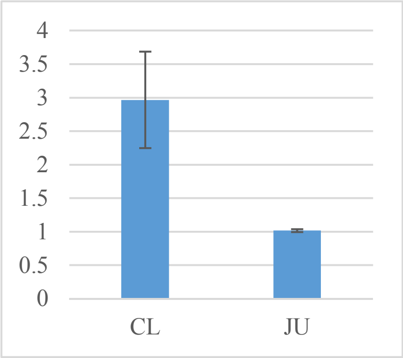

K1QFS1, Collagen alpha-6(VI) chain
CL-high, $p < 0.05$

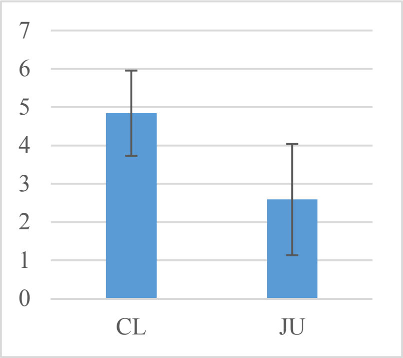

K1R3E8, Collagen alpha-5(VI) chain
No variation, $p > 0.05$

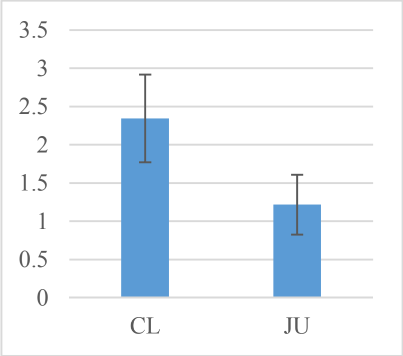

K1PDA4, Collagen alpha-4(VI) chain
CL-high, $p < 0.05$

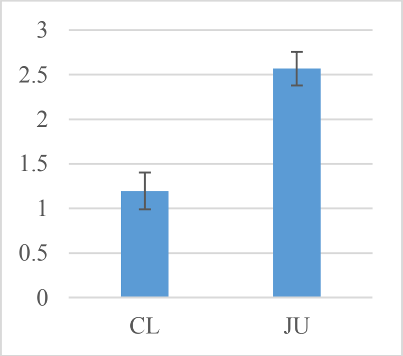

K1R0Q6, Laminin subunit alpha
JU-high, $p < 0.05$

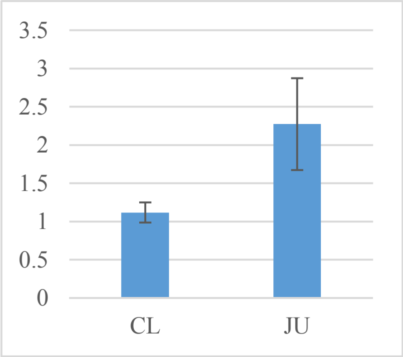

K1PTA3, Collagen alpha-4(VI) chain
JU-high, $p < 0.05$

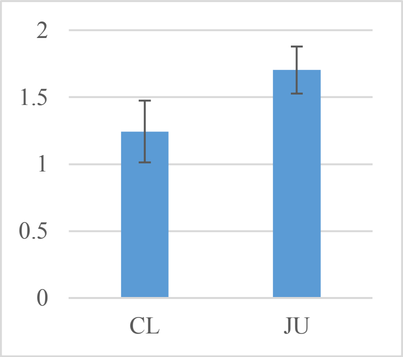

K1QWZ6, Dolichyl-diphosphooligosaccharide--protein glycosyltransferase subunit 1
No variation, $p > 0.05$

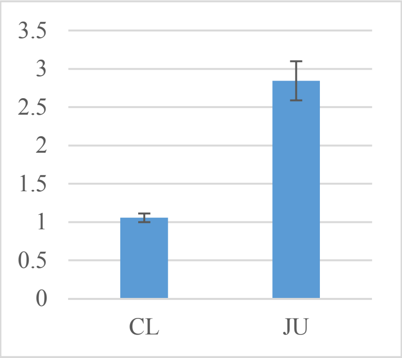

K1R6E6, Basement membrane-specific heparan sulfate proteoglycan core protein
JU-high, $p < 0.05$

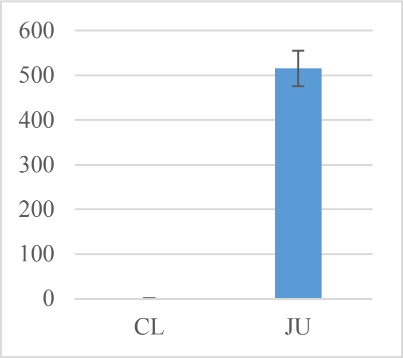

K1PPU7, Hemicentin-1
JU-high, $p < 0.05$

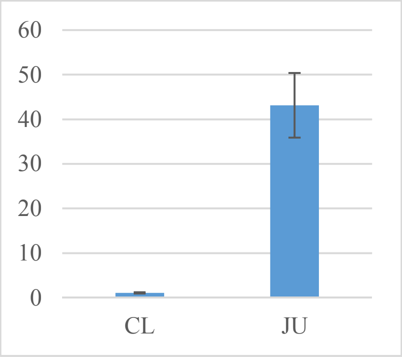

K1PZ82, Collagen alpha-3(VI) chain
JU-high, $p < 0.05$

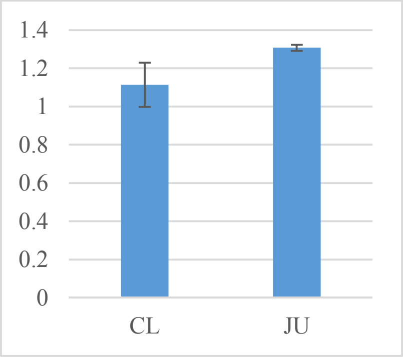

K1QHM2, Dolichyl-diphosphooligosaccharide--protein glycosyltransferase subunit 2
JU-high, $p < 0.05$

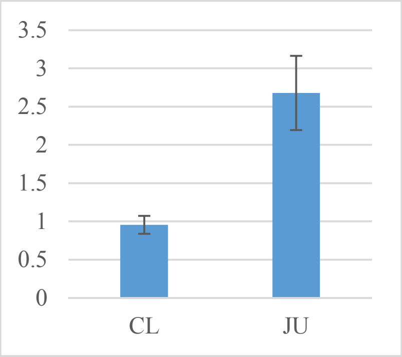

K1Q4S5, Cadherin-87A
JU-high, $p < 0.05$

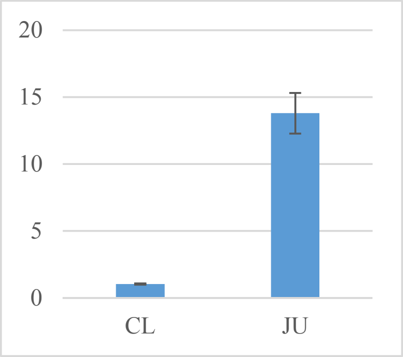

K1R289, Collagen alpha-3(VI) chain
JU-high, $p < 0.05$

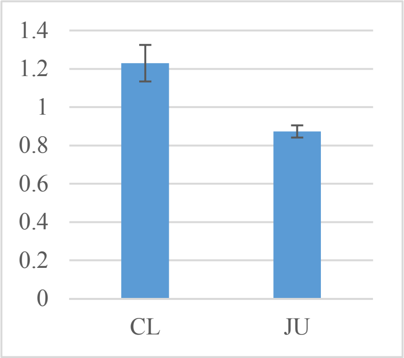

K1QQV6, Collagen alpha-1(XII) chain
CL-high, $p < 0.05$

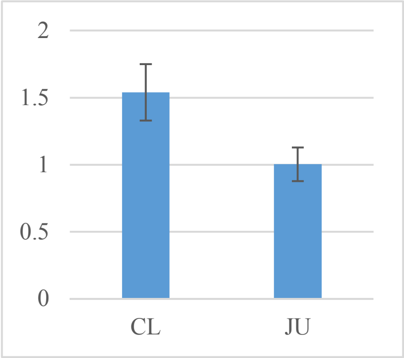

K1R3T3, Transcription factor BTF3-like protein 4
CL-high, $p < 0.05$

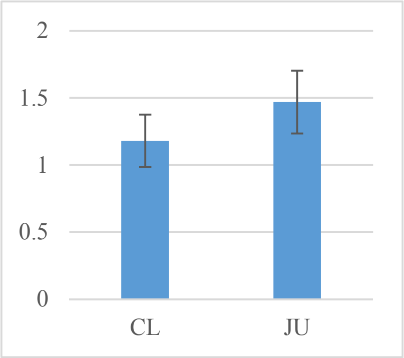

K1PM66, 60S ribosomal protein L12
No variation, $p > 0.05$

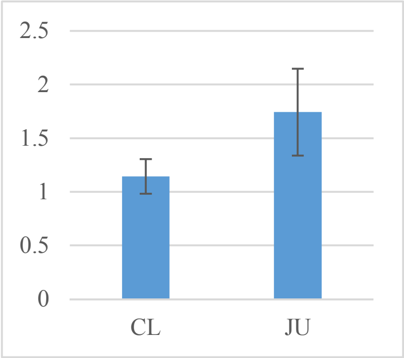

K1PQP2, Nucleolin
No variation, $p > 0.05$

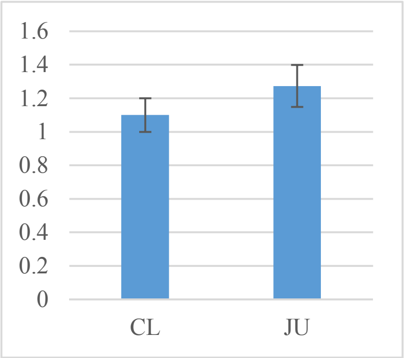

K1Q2L2, 60S ribosomal protein L12
No variation, $p > 0.05$

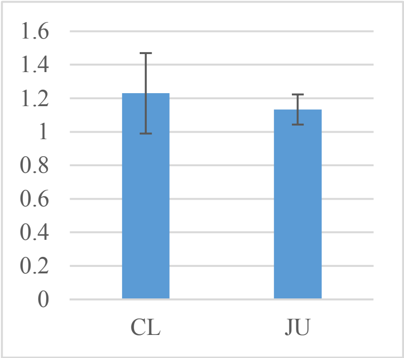

A7M7T7, Non-selenium glutathione peroxidase
No variation, $p > 0.05$

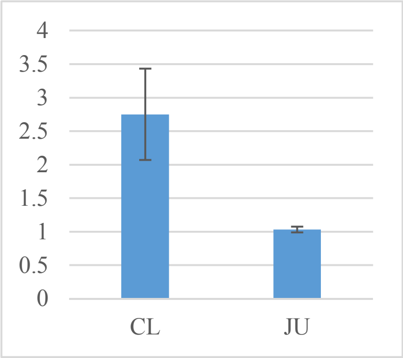

K1R7F8, Peroxiredoxin-5, mitochondrial
CL-high, $p < 0.05$

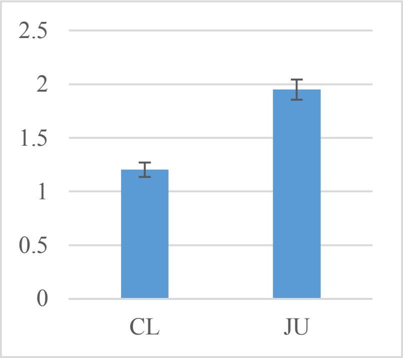

K1PWQ27, 60 kDa neurofilament protein
JU-high, $p < 0.05$

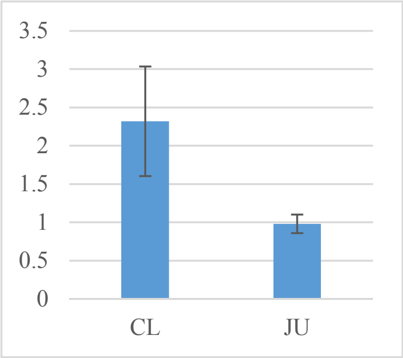

K1Q2X5, Synaptopodin-2
CL-high, $p < 0.05$

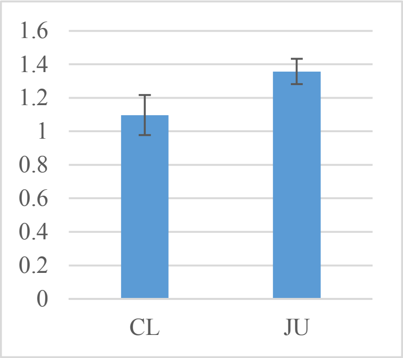

K1QRU8 9, Myosin heavy chain, striated muscle
JU-high, $p < 0.05$

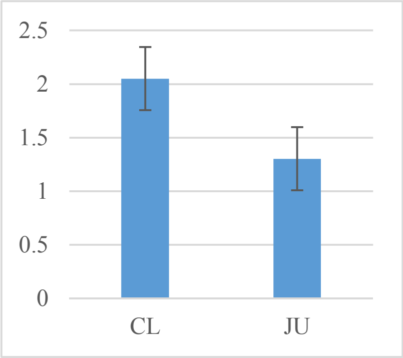

K1Q801, Myosin regulatory light chain A, smooth adductor muscle
CL-high, $p < 0.05$

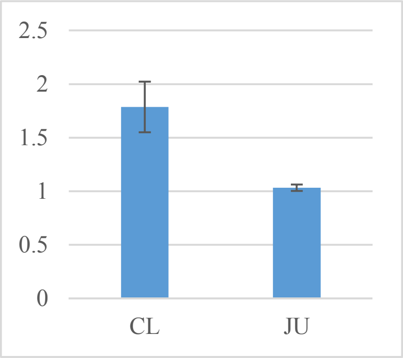

K1PAG1, Dynein beta chain, ciliary
CL-high, $p < 0.05$

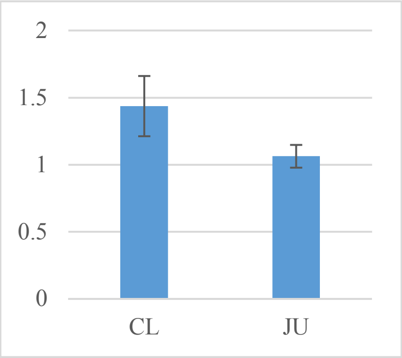

K1PX83, Dynein heavy chain 5, axonemal
No variation, $p > 0.05$

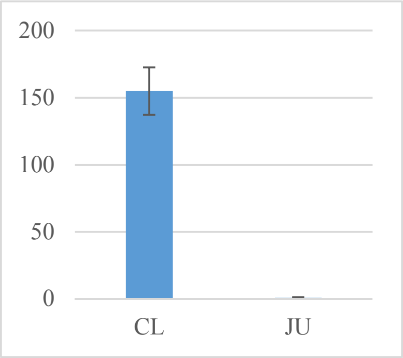

K1QM96, Chymotrypsin B
CL-high, $p < 0.05$

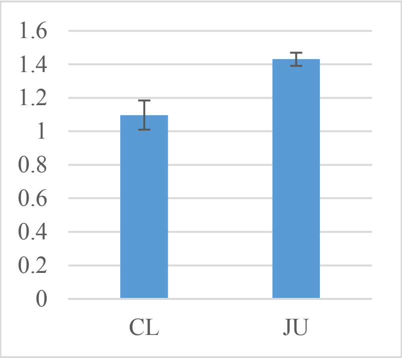

K1R712, Transforming growth factor-beta receptor-associated protein 1
JU-high, $p < 0.05$

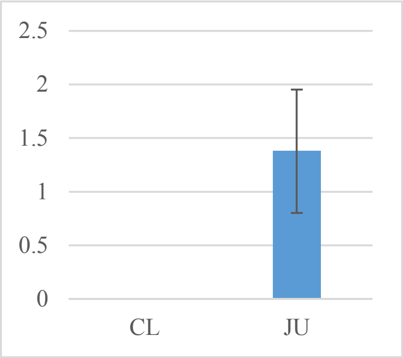

K1R6J8, Wnt inhibitory factor 1
Only detected in JU.

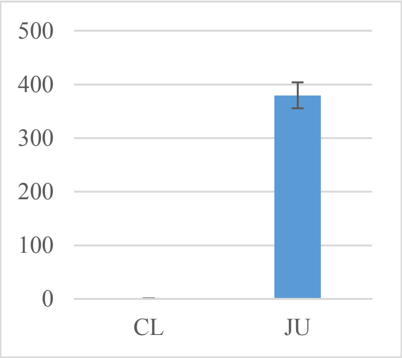

K1PHQ7, Follistatin-related protein 4
JU-high, $p < 0.05$

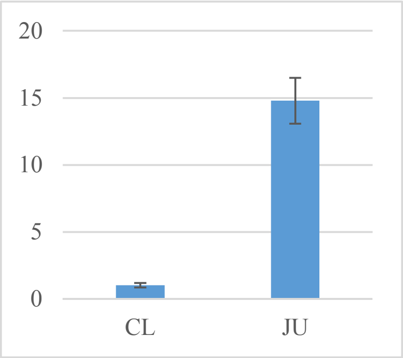

K1QX16, Tyrosine-protein phosphatase Lar
JU-high, $p < 0.05$
